# Promoter methylation changes and vascular dysfunction in pre-eclamptic umbilical vein

**DOI:** 10.1186/s13148-019-0685-2

**Published:** 2019-05-28

**Authors:** Qinqin Gao, Xiaorong Fan, Ting Xu, Huan Li, Yun He, Yuxian Yang, Jie Chen, Hongmei Ding, Jianying Tao, Zhice Xu

**Affiliations:** 1 Institute for Fetology and Department of Obstetrics and Gynecology, First Hospital of Soochow University, Suzhou, 215006 China; 2 Center for Perinatal Biology, Loma Linda University, California, USA; 3 Department of Obstetrics and Gynecology, Suzhou Municipal Hospital, Suzhou, China; 4 Department of Obstetrics and Gynecology, Affiliated Suzhou Hospital of Nanjing University of Chinese Medicine, Suzhou, China

**Keywords:** Pre-eclampsia, Arginine vasopressin, Oxytocin, DNA methylation, Umbilical vein dysfunction

## Abstract

**Background:**

Hypertension is one of primary clinical presentations of pre-eclampsia. The occurrence and progress of hypertension are closely related to vascular dysfunction. However, information is limited regarding the pathological changes of vascular functions in pre-eclamptic fetuses. Human umbilical cord vein was used to investigate the influence of pre-eclampsia on fetal blood vessels in this study.

**Results:**

The present study found that the vasoconstriction responses to arginine vasopressin (AVP) and oxytocin (OXT) were attenuated in the pre-eclamptic umbilical vein as compared to in normal pregnancy, which was related to the downregulated AVP receptor 1a (AVPR1a), OXT receptor (OXTR), and protein kinase C isoform β (PKCβ), owing to the deactivated gene transcription, respectively. The deactivated *AVPR1a*, *OXTR*, and *PKCB* gene transcription were respectively linked with an increased DNA methylation within the gene promoter.

**Conclusions:**

To the best of our knowledge, this study first revealed that a hyper-methylation in gene promoter, leading to relatively reduced patterns of AVPR1a, OXTR, and PKCB expressions, which was responsible for the decreased sensitivity to AVP and OXT in the umbilical vein under conditions of pre-eclampsia. The data offered new and important information for further understanding the pathological features caused by pre-eclampsia in the fetal vascular system, as well as roles of epigenetic-mediated gene expression in umbilical vascular dysfunction.

## Background

Pre-eclampsia (PE) is a leading cause of maternal morbidity, mortality, and premature birth in both developed and developing countries [1, 2]. Although PE in women is a multi-systemic syndrome with unknown etiology, hypertension is a primary clinical presentation of PE. As a surrogate end point for vascular risk, vascular dysfunction is closely related to the occurrence and progress of hypertension. However, information regarding the pathological changes of vascular functions in pre-eclamptic fetuses is limited. The umbilical cord is a conduit between the developing fetus and placenta. Umbilical cord vessels are primary vascular structures that may reflect problems originated from inadequate changes in maternal and fetal vascular systems. Human umbilical cord normally contains two arteries and one vein. The umbilical vein supplies the fetus with oxygenated, nutrient-rich blood from the placenta. Vascular functions of the umbilical vein are so important for placental-fetal circulation and fetal development in utero. Therefore, the present study was conducted with umbilical veins from healthy and pre-eclamptic pregnancy to investigate whether and how vascular functions would be affected under conditions of PE.

Because umbilical vessels have no autonomic innervation [3, 4], circulating and locally synthesized vasoactive substances are important in controlling vascular functions and blood flow in the placental-fetal circulation. As stress hormones, arginine vasopressin (AVP) and oxytocin (OXT) are mainly synthesized in the magnocellular neurons of the paraventricular and supraoptic nucleus of the hypothalamus. In most vascular beds, AVP and OXT are potent vasoconstrictors [5]. AVP has long been implicated in controlling blood pressure and vascular tone through binding of smooth muscle receptors (mainly classified into V1a (AVPR1a), V1b (AVPR1b), and V2 (AVPR2) subtypes) [6–8]. In normal delivery, high AVP concentrations in human umbilical cord blood have been reported [9, 10]. Similarly, oxytocin (OXT), a nine amino acid neuropeptide, is also increased at late pregnancy and onset of labor [11, 12]. The actions of both central and peripheral OXT are mediated through oxytocin receptor (OXTR) [13]. It has been reported that AVP- and OXT-induced vasocontractions are mainly regulated by protein kinase C (PKC) pathway [6, 14, 15].

In humans, high AVP and OXT concentrations are demonstrated in umbilical cord blood during normal delivery [9, 10, 12]. Do the high AVP and OXT in the circulation cause remarkable vasoconstrictions in umbilical vessels? Would AVP and OXT play the same physiological roles in pre-eclamptic umbilical vessels as they do in the normal ones? In fact, such is the paucity of knowledge of vascular reactivities of the umbilical vein, with very limited studies and information on umbilical vascular functions and none has compared umbilical vascular responses of AVP and OXT between PE and normal pregnancy. The present study, therefore, investigated the contractile responses of AVP and OXT in normal and pre-eclamptic umbilical vein, to reveal special features of umbilical vascular regulations and possible pathophysiological changes, as well as its underlying mechanisms under PE condition. The data gained in the present study provided new and critical information on regulations of umbilical vascular functions under pre-eclamptic conditions that in favor of further understanding the pathological features and mechanisms of PE as well as vascular diseases in fetal origins.

## Results

### AVP or OXT-induced contractions in human umbilical vein

**Fig. 1:**
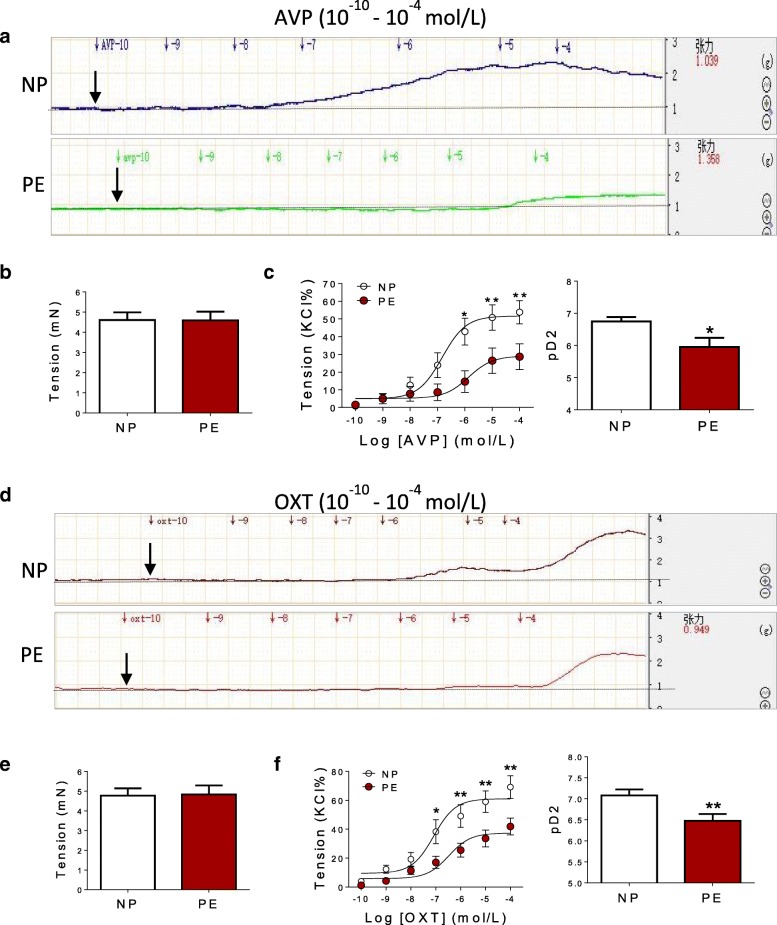
AVP- and OXT-mediated vascular reactivity in human umbilical vein. **a**, **c** Concentration-response curves of AVP-induced dose-dependent contractions in HUV (*N* = 20, *n* = 38 each group). **b**, **e** KCl-induced maximal contractions in HUV (*N* = 20, *n* = 38 each group). **d**, **f** Concentration-response curves of OXT-induced dose-dependent contractions in HUV (*N* = 21, *n* = 36 each group). AVP arginine vasopressin, OXT oxytocin, KCl potassium chloride, NP normal pregnancy, PE pre-eclampsia, HUV human umbilical vein. Error bars denote SEM. *P < 0.05; **P < 0.01. *N* number of participants, *n* number of HUV rings

Both AVP and OXT could induce dose-dependent constrictions in human umbilical vein (HUV) (Fig. 1a, d). There were no significant differences in KCl-induced maximal contraction between NP and PE group (Fig. 1b, e), whereas, the Emax (AVP- or OXT-induced contraction at 10^−4^ mol/L) and pD2 (−log[50% effective concentration]) values for AVP and OXT were significantly decreased in pre-eclamptic umbilical vein (Fig. 1c, f). These data indicated that pre-eclamptic umbilical vein was significantly insensitive to AVP and OXT.

### Expression of AVP or OXT receptors in human umbilical vein

**Fig. 2:**
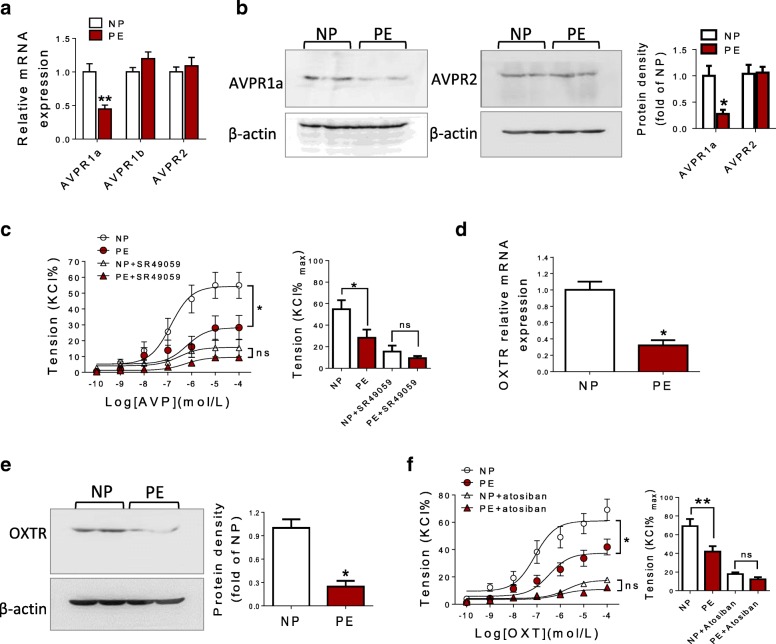
Expression of AVP and OXT receptors in human umbilical vein. **a**, **b** mRNA and protein levels of AVP receptors in HUV were determined by qRT-PCR and Western blot. **c** Effects of SR49059 on AVP-mediated vasoconstrictions in HUV (*N* = 13, *n* = 25 each group). **d**, **e** mRNA and protein levels of OXT receptor in HUV. **f** Effects of atosiban on OXT-mediated vasoconstrictions in HUV (*N* = 14, *n* = 28 each group). SR49059, AVPR1a-specific antagonist; Atosiban, OXT-specific antagonist. Error bars denote SEM. *P < 0.05; **P < 0.01; ns no significances, *N* number of participants, *n* number of HUV rings

In the vasculature, AVP receptors include AVPR1a, AVPR1b, and AVPR2 [7]. Compared with NP, mRNA and protein levels of AVPR1a, not AVPR2, were decreased in the PE group (Fig. 2a, b). SR49059 (AVPR1a-specific antagonist) completely blocked AVP-mediated contractions in both NP and PE groups and without significant differences in AVP-induced vasoconstrictions between the two groups after pretreatment with SR49059 (Fig. 2c). Similarly, as shown in Fig. 2d and e, there was a significant decrease in mRNA and protein of OXTR in the PE group. Meanwhile, OXTR-specific antagonist (atosiban) could completely block OXT-mediated contractions in the umbilical vein, without significant differences between NP and PE groups after pretreated with atosiban (Fig. 2f). These data indicated that the decreased sensitivity of pre-eclamptic umbilical vein to AVP and OXT was related to the downregulated AVPR1a and OXTR due to the deactivated gene transcription, respectively.

### The decreased sensitivity of AVP and OXT was also dependent on PKC pathway

**Fig. 3:**
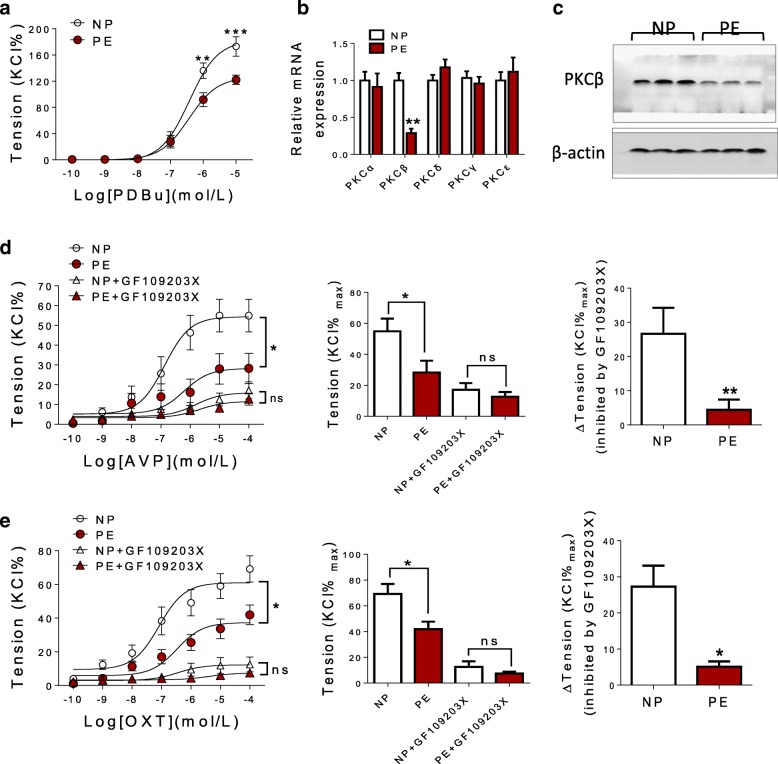
The decreased sensitivity of AVP and OXT was dependent on PKC pathway. **a** PDBu induced vasoconstrictions in HUV (*N* = 12, *n* = 26 each group). **b** The mRNA levels of PKCα, PKCβ, PKCγ, PKCδ, and PKCε in HUV determined by qRT-PCR (*N* = 40 each group). **c** The protein levels of PKCβ in HUV determined by Western blot. **d** The inhibitory effect of GF109203X on AVP-induced contractions in HUV (*N* = 12, *n* = 19 each group). **e** The inhibitory effect of GF109203X on OXT-induced contractions in HUV (*N* = 12, *n* = 18 each group). GF109203X, PKC antagonist; PDBu, Phorbol 12, 13-dibutyrate (PKC activator). Error bars denote SEM. *P < 0.05; **P < 0.01; ***P < 0.001; ns no significances, *N* number of participants, *n* number of HUV rings

AVP- and OXT-induced vasocontractions are mainly regulated by PKC pathway [6, 14, 15]. As shown in Fig. 3a, PKC agonist (PDBu) caused weaker dose-dependent contractions in pre-eclamptic HUV than that of NP group. In the vasculature, PKC mainly includes α, β, γ, δ, and ε isoforms [16]. There were no significant differences in PKCα, PKCγ, PKCε, and PKCδ mRNA expression between NP and PE group; however, mRNA levels of PKCβ were significantly decreased in PE compared with that in NP group (Fig. 3b). Protein levels of PKCβ were also significantly decreased in pre-eclamptic HUV (Fig. 3c). Meanwhile, PKC-specific antagonist (GF109203X) could restrain AVP- or OXT-induced vasoconstrictions in both NP and PE groups, without significant differences in AVP- or OXT-mediated vasoconstrictions between NP and PE group following pretreatment with GF109203X (Fig. 3d, e). Meanwhile, GF109203X could produce a weaker inhibitory effect on AVP- or OXT-mediated vasoconstrictions in NP group (Fig. 3d, e). These data indicated that the decreased sensitivity of pre-eclamptic umbilical vein to AVP and OXT was also related to the downregulated PKC pathway.

### DNA methylation of CpG locus within *AVPR1a* gene promoter in human umbilical vein

**Fig. 4:**
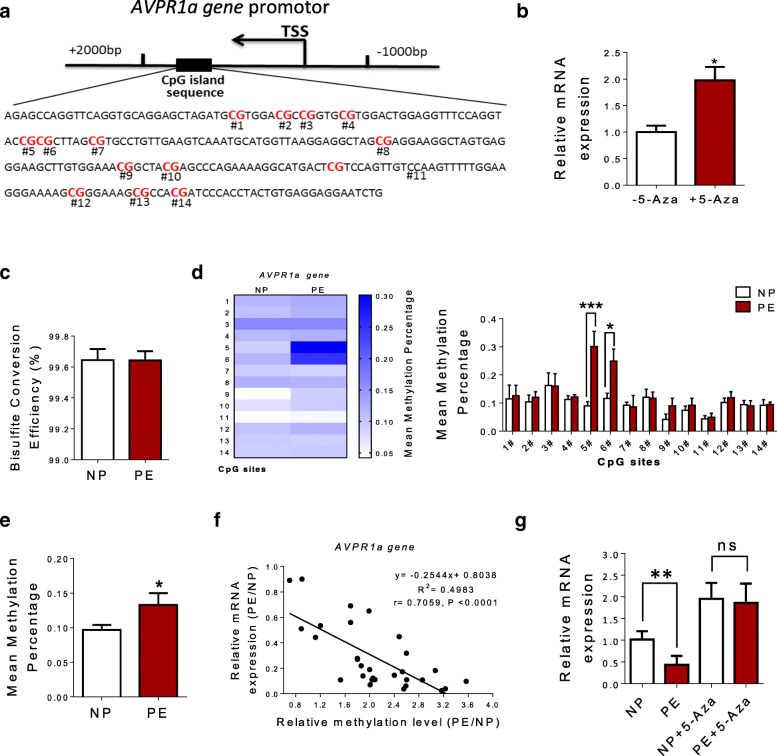
DNA methylation of CpG locus at *AVPR1a* gene promoter in human umbilical vein. **a** Bioinformatic analysis of CpG islands of *AVPR1a* gene from upstream − 1.5 kb to downstream + 1.5 kb region. Sequence analysis identified one CpG island in exon that contains 14 CpG sites, located at positions + 1292 to + 1484 from the translation start site (TSS, defined as position 1) in *AVPR1a* gene promoter. **b** The mRNA levels of *AVPR1a* in HUVECs after treatment with 5-Aza-2′-deoxycytidine (5-Aza) for 2 days. **c** Represent image of bisulfite conversion efficiency between NP and PE group (*N* = 30 each group). **d**–**e** The mean methylation status of CpG locus (the total and each tested) at AVPR1a gene promoter in HUV (*N* = 30 each group). **f** Expression analysis of *AVPR1a* gene and its correlation with methylation levels of CpG sites (5 and 6). DNA methylation/mRNA correlation plots for *AVPR1a* gene identified by causal inference test (*N* = 30 each group). The *Y*-axis represents the relative expression level of *AVPR1a* gene which was detected with qRT-PCR method. The *X*-axis represents the relative mean methylation level of the CpG sites (5 and 6) at AVPR1a gene promoter in HUV. r: Pearson correlation coefficient. **g** mRNA levels of AVPR1a gene in normal and pre-eclamptic HUVECs after 5-Aza treatment (*N* = 8 each group). Error bars denote SEM. *P < 0.05; **P < 0.01; ***P < 0.001. *N* number of participants

*AVPR1a* is located on chromosome 12q14.2. To clarify whether the deactivated transcription of *AVPR1a* was associated with DNA methylation alterations, we assessed changes of *AVPR1a* transcription after adding 5-Aza-2′-deoxycytidine (5-Aza, a specific DNA methylation transferase inhibitor) in human umbilical cord vein endothelial cells (HUVECs). In HUVECs, 5-Aza treatment significantly increased *AVPR1a* gene transcription (Fig. 4b). One CpG island contains 14 CpG sites within exon of *AVPR1a* gene (Fig. 4a). Table 1 showed CpG labels. Next, we validated methylation levels of these 14 CpG sites by targeted bisulfite sequencing. The bisulfite conversion rate of each sample was higher than 99%, and no significant difference was observed between NP and PE group, indicating bisulfite conversion was efficient and reliable in the experiments (Fig. 4c). Compared with NP, the mean methylation percentage of these 14 CpG sites in pre-eclamptic umbilical vein was significantly increased with specific CpG site 5 and 6 (Fig. 4d–e, Table 2). Correlation analysis between *AVPR1a* gene methylation and expression was also conducted. There was a significantly inverse correlation between the methylation statuses of CpG sites (5 and 6) in *AVPR1a* gene promoter and *AVPR1a* gene expression (Fig. 4f). In normal and pre-eclamptic HUVECs, after 5-Aza treatment, mRNA levels of *AVPR1a* were significantly increased and without significant differences between the two groups (Fig. 4g).

**Table 1:** Methylated CpG sites at *AVPR1a* gene promoter measured in this study

Gene	Position	Genomic location	Relative to TSS, bp
*AVPR1a*	1	Chr12: 63545106	+ 1484
	2	Chr12: 63545111	+ 1479
	3	Chr12: 63545119	+ 1471
	4	Chr12: 63545152	+ 1438
	5	Chr12: 63545174	+ 1416
	6	Chr12: 63545180	+ 1410
	7	Chr12: 63545212	+ 1378
	8	Chr12: 63545251	+ 1339
	9	Chr12: 63545258	+ 1332
	10	Chr12: 63545260	+ 1330
	11	Chr12: 63545284	+ 1306
	12	Chr12: 63545289	+ 1301
	13	Chr12: 63545292	+ 1298
	14	Chr12: 63545298	+ 1292

**Table 2:** The methylation status of CpG locus (the total and each tested) at *AVPR1a* gene promoter. The data was expressed as mean ± SEM. *PE* pre-eclampsia, *NP* normal pregnant. **P < 0.01; ***P < 0.001

Gene	Position	NP	PE
*AVPR1a*	1	0.104 ± 0.028	0.123 ± 0.028
	2	0.107 ± 0.032	0.119 ± 0.021
	3	0.165 ± 0.027	0.163 ± 0.010
	4	0.115 ± 0.015	0.122 ± 0.009
	5	0.094 ± 0.015	0.291 ± 0.064**
	6	0.112 ± 0.019	0.280 ± 0.066***
	7	0.092 ± 0.039	0.083 ± 0.014
	8	0.131 ± 0.028	0.111 ± 0.031
	9	0.047 ± 0.019	0.088 ± 0.026
	10	0.072 ± 0.018	0.088 ± 0.028
	11	0.043 ± 0.011	0.049 ± 0.016
	12	0.096 ± 0.019	0.131 ± 0.015
	13	0.102 ± 0.040	0.100 ± 0.034
	14	0.094 ± 0.021	0.100 ± 0.004
	Average	0.098 ± 0.013	0.132 ± 0.017*

### DNA methylation of CpG locus within *OXTR* gene promoter in human umbilical vein

**Fig. 5:**
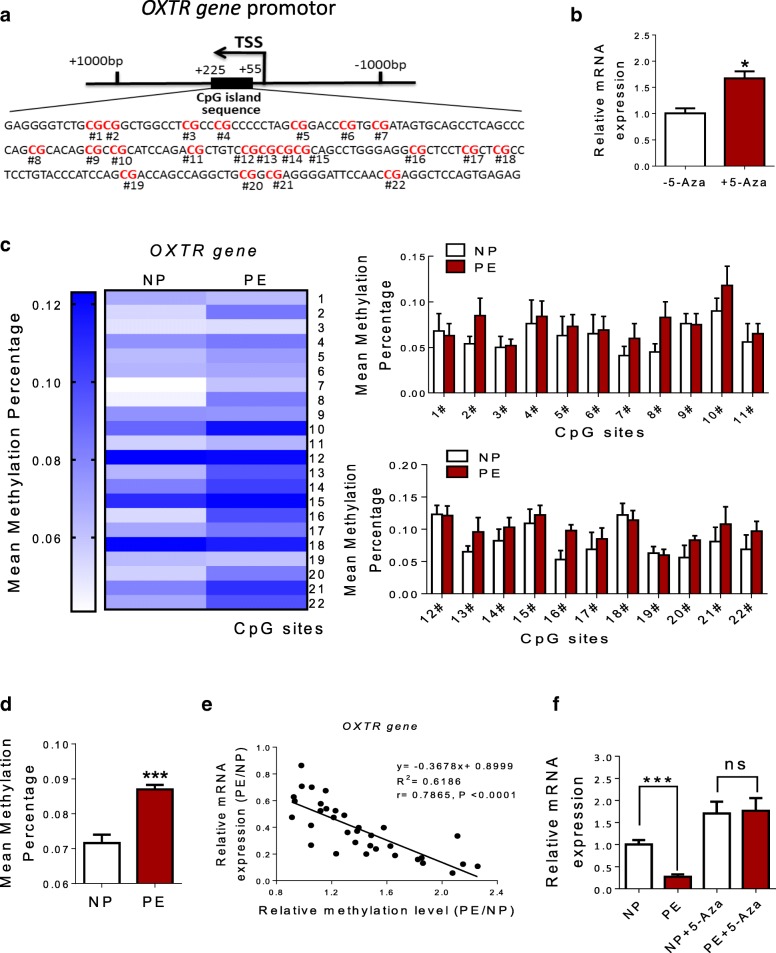
DNA methylation of CpG locus at OXTR gene promoter in human umbilical vein. **a** Sequence analysis identified one CpG island in exon 1 that contains 22 CpG sites, located at positions + 55 to + 225 from the TSS in the *OXTR* gene promoter. **b** mRNA levels of *OXTR* in HUVECs after treatment with 5-Aza. **c**–**d** Represent the mean methylation status of the genomic regions in OXTR gene promoter. Each bar represents mean methylation percentage in a genomic region of a sample. **e** Expression analysis of OXTR gene and its correlation with methylation levels of 22 CpG sites. DNA methylation/mRNA correlation plots for OXTR gene identified by causal inference test. The *y*-axis represents the relative expression level of OXTR gene which was detected with qRT-PCR method. The *x*-axis represents the relative mean methylation level of all the 22 CpG sites in OXTR gene. r: Pearson correlation coefficient. **f** mRNA levels of OXTR gene in normal and pre-eclamptic HUVECs after 5-Aza treatment (*N* = 8 each group). Error bars denote SEM.*P < 0.05; ***P < 0.001. *N* number of participants

*OXTR* is located on chromosome 3p25. Sequence analysis identified one CpG island that contains 22 CpG sites within exons of the *OXTR* gene (Fig. 5a). Table 3 provided a key for CpG labels. In HUVECs, after 5-Aza treatment, OXTR mRNA level was significantly increased (Fig. 5b). Compared with NP, mean methylation percentage of the total 22 CpG sites in the PE group was remarkably increased (Fig. 5d), whereas, no significant differences were observed in each tested CpG site between NP and PE group (Fig. 5c, d). Table 4 showed the position and methylation levels of these 22 CpG sites. As shown in Fig. 5e, there was a significantly inverse correlation between the methylation status of 22 CpG sites in *OXTR* gene promoter and *OXTR* gene expression. In normal and pre-eclamptic HUVECs, mRNA levels of *OXTR* were significantly increased, and no significant differences were observed between the two groups after 5-Aza treatment (Fig. 5g).

**Table 3:** Methylated CpG sites at *OXTR* gene promoter measured in this study

Gene	Position	Genomic location	Relative to TSS, bp
*OXTR*	1	Chr3: 8811075	+ 225
	2	Chr3: 8811090	+ 210
	3	Chr3: 8811093	+ 207
	4	Chr3: 8811108	+ 192
	5	Chr3: 8811128	+ 172
	6	Chr3: 8811132	+ 168
	7	Chr3: 8811139	+ 161
	8	Chr3: 8811153	+ 147
	9	Chr3: 8811155	+ 145
	10	Chr3: 8811157	+ 143
	11	Chr3: 8811159	+ 141
	12	Chr3: 8811166	+ 134
	13	Chr3: 8811176	+ 124
	14	Chr3: 8811179	+ 121
	15	Chr3: 8811186	+ 114
	16	Chr3: 8811209	+ 91
	17	Chr3: 8811213	+ 87
	18	Chr3: 8811219	+ 81
	19	Chr3: 8811229	+ 71
	20	Chr3: 8811233	+ 67
	21	Chr3: 8811243	+ 57
	22	Chr3: 8811245	+ 55

**Table 4:** The methylation status of CpG locus (the total and each tested) at *OXTR* gene promoter. The data was expressed as mean ± SEM. *PE* pre-eclampsia, *NP* normal pregnant. ***P < 0.001

Gene	Position	NP	PE
*OXTR*	1	0.068 ± 0.019	0.063 ± 0.013
	2	0.054 ± 0.008	0.085 ± 0.019
	3	0.050 ± 0.012	0.052 ± 0.007
	4	0.076 ± 0.026	0.084 ± 0.017
	5	0.063 ± 0.021	0.073 ± 0.013
	6	0.065 ± 0.021	0.069 ± 0.015
	7	0.041 ± 0.010	0.060 ± 0.016
	8	0.045 ± 0.004	0.083 ± 0.017
	9	0.076 ± 0.011	0.075 ± 0.012
	10	0.090 ± 0.014	0.118 ± 0.021
	11	0.056 ± 0.020	0.065 ± 0.011
	12	0.123 ± 0.014	0.121 ± 0.015
	13	0.065 ± 0.009	0.096 ± 0.022
	14	0.082 ± 0.018	0.103 ± 0.015
	15	0.109 ± 0.022	0.122 ± 0.015
	16	0.053 ± 0.014	0.098 ± 0.009
	17	0.069 ± 0.026	0.085 ± 0.017
	18	0.122 ± 0.018	0.114 ± 0.015
	19	0.063 ± 0.010	0.060 ± 0.009
	20	0.056 ± 0.019	0.083 ± 0.007
	21	0.081 ± 0.022	0.108 ± 0.027
	22	0.069 ± 0.022	0.097 ± 0.015
	Average	0.0716 ± 0.0024	0.0870 ± 0.0013***

### DNA methylation of CpG locus within *PKCΒ* gene promoter in human umbilical vein

**Fig. 6:**
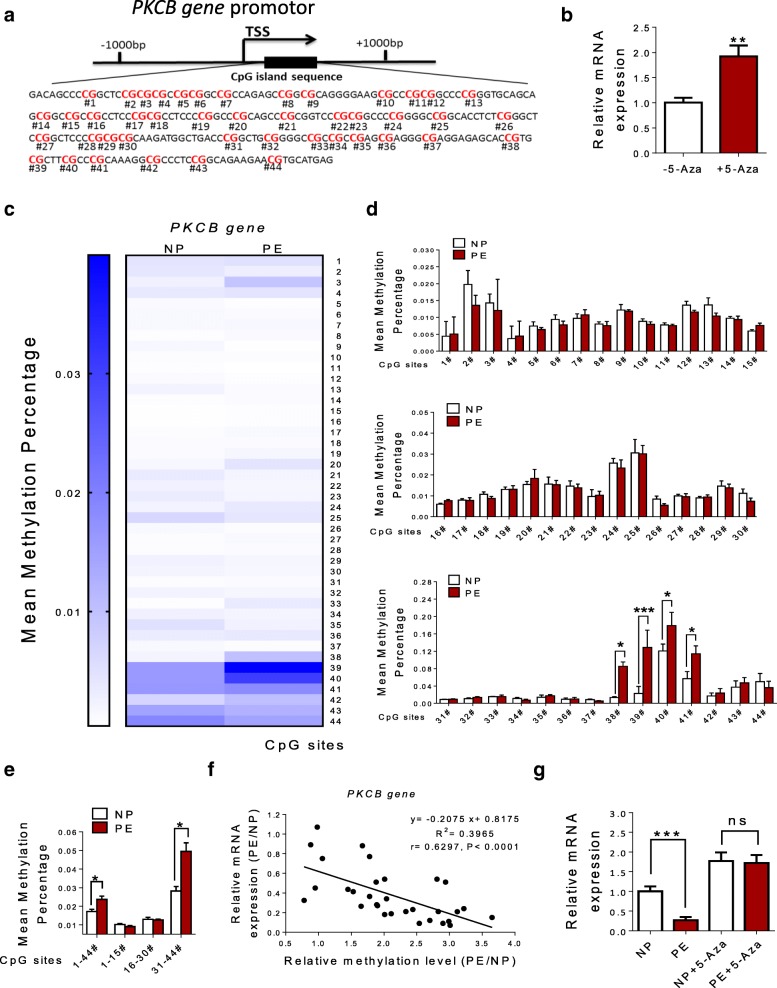
DNA methylation of CpG locus at *PKCΒ* gene promoter in human umbilical vein. **a** One CpG island that contains 44 CpG sites, located at positions + 27 to + 321 from TSS at *PKCΒ* gene promoter. **b** mRNA levels of *PKCΒ* in HUVECs after treatment with 5-Aza. **c**–**e** The mean methylation status of CpG locus (the total and each tested) at *PKCΒ* gene promoter in HUV (*N* = 30 each group). **f** DNA methylation/mRNA correlation plots for *PKCΒ* gene identified by causal inference test (*N* = 30 each group). **g** mRNA levels of OXTR gene in normal and pre-eclamptic HUVECs after 5-Aza treatment (*N* = 8 each group). Error bars denote SEM.*P < 0.05; **P < 0.01; ***P < 0.001. *N* number of participants

*PKCΒ* is located on chromosome 16p12.2. One CpG island contains 44 CpG sites within exons of *PKCΒ* gene (Fig. 6a). 5-Aza treatment also significantly increased *PKCΒ* gene transcription in HUVECs (Fig. 6b). Targeted bisulfite sequencing showed that compared with NP, the mean methylation percentage of the total 44 CpG sites was remarkably increased with specific CpG sites (38–41) within *PKCΒ* gene promoter in the PE group, whereas no significant difference was observed in other specific CpG sites between NP and PE group (Fig. 6c–e). Position and methylation levels of the 44 CpG sites were listed in Table 5. After careful analysis of DNA methylation and expression data, it is concluded that there was also a significantly inverse correlation between the methylation statuses of 38–41 CpG sites and *PKCΒ* expression (Fig. 6f). After 5-Aza treatment, mRNA levels of *PKCΒ* were significantly increased, and no significant differences were observed between normal and pre-eclamptic HUVECs (Fig. 6g).

**Table 5:** The methylation status of CpG locus (the total and each tested) at *PKCΒ* gene promoter. The data was expressed as mean ± SEM. *PE* pre-eclampsia, *NP* normal pregnant. *P < 0.05; **P < 0.01; ***P < 0.001

Gene	Position	NP	PE
*PKCB*	31	0.009 ± 0.0003	0.007 ± 0.0008
	32	0.009 ± 0.0013	0.011 ± 0.0013
	33	0.010 ± 0.0008	0.009 ± 0.0007
	34	0.014 ± 0.0003	0.013 ± 0.0007
	35	0.008 ± 0.0006	0.011 ± 0.0018
	36	0.007 ± 0.0008	0.008 ± 0.0009
	37	0.008 ± 0.0006	0.009 ± 0.0007
	38	0.016 ± 0.0048	0.084 ± 0.0156*
	39	0.021 ± 0.0095	0.108 ± 0.0166***
	40	0.121 ± 0.0696	0.186 ± 0.0536*
	41	0.063 ± 0.0234	0.134 ± 0.0237**
	42	0.014 ± 0.0018	0.014 ± 0.0016
	43	0.028 ± 0.0117	0.043 ± 0.0178
	44	0.008 ± 0.0011	0.008 ± 0.0010
	Average	0.024 ± 0.0038	0.046 ± 0.0096**

## Discussion

This present study found a special feature of AVP- and OXT-mediated vascular contractions in pre-eclamptic umbilical vasculature. The main findings are as follows: (1) Compared with the normal control, the vasoconstriction responses to AVP and OXT were attenuated in pre-eclamptic umbilical vein, which was related to the downregulated AVPR1a, OXTR, and PKCB, owing to the deactivated gene transcription, respectively. (2) The deactivated *AVPR1a*, *OXTR*, and *PKCB* transcriptions were respectively linked with an increased DNA methylation within gene promoter. The data gained not only offered new information for further understanding the pathological features and mechanisms of pre-eclamptic umbilical cords, but also providing novel clues for roles of epigenetic-mediated gene expression in fetal vascular dysfunction.

Although it is well known that AVP and OXT can produce vascular contractions in adults [5, 17], data regarding their functional effects on fetal blood vessels is limited. In human, umbilical vessels are only healthy fetal blood vessels that can be obtained ethically in medical studies. Both of AVP and OXT exhibited significant dose-dependent vasoconstrictions in human fetal umbilical vein, suggesting that the two peptides are critically involved in the regulating umbilical vascular tone and circulation. Notably, we found that compared with normal pregnancy, pre-eclamptic umbilical vein was significantly insensitive to AVP and OXT, which was not only associated with their respective receptors, but also correlated with PKC pathway. This finding was supported by the following data: (1) The mRNA and protein levels of AVPR1a and OXTR were remarkably decreased in pre-eclamptic umbilical vein. Meanwhile, AVPR1a- or OXTR-specific antagonist could completely block AVP- and OXT-mediated contractions in umbilical vein, respectively. (2) PKC agonist caused weaker dose-dependent contractions, and PKC antagonist produced a weaker inhibitory effect on AVP- and OXT-mediated vasoconstrictions in pre-eclamptic umbilical vein; furthermore, mRNA and protein levels of PKCβ were significantly decreased in pre-eclamptic umbilical vein. These data above indicated that the decreased sensitivity of pre-eclamptic umbilical vein to AVP and OXT was related to the downregulated AVPR1a, OXTR, and PKCB, particularly with their deactivated gene transcription.

The number of studies in humans and laboratory animals indicated promoter DNA methylation levels are important for transcriptional regulation of *AVPR1a* [18–20], *OXTR* [21–23], and *PKCB* [24–27]. In exploring the possible underlying mechanisms of the altered AVP- and OXT-mediated vascular functions, the present study also focused on epigenetic causes. Firstly, to clarify whether the deactivated transcriptions of *AVPR1a*, *OXTR*, and *PKCB* are owing to DNA methylation, we assessed the changes of these gene transcriptions after adding 5-Aza in HUVECs. 5-Aza treatment significantly increased *AVPR1a*, *OXTR*, and *PKCB* gene transcriptions, indicating that these gene transcriptions were regulated by DNA methylation in human umbilical vascular cells. Secondly, we evaluated DNA methylation status of CpG sites within *AVPR1a*, *OXTR*, and *PKCB* gene promoter and found that the mean methylation percentages of CpG sites within CpG islands in *AVPR1a*, *OXTR*, and *PKCB* gene promoter were obviously increased in umbilical vein under conditions of PE. Thirdly, we conducted correlation analysis between gene methylation and expression and found that there was a significantly inverse correlation between DNA methylation levels of gene promoter and gene transcription. Fourthly, we isolated and cultured HUVECs in vitro and evaluated expressions of these genes in both of normal and pre-eclamptic HUVECs after 5-Aza treatment. Compared with normal, mRNA levels of these genes were decreased in the pre-eclamptic HUVECs. After 5-Aza treatment, mRNA levels of these genes were significantly increased in both of normal and pre-eclamptic HUVECs, and no significant differences were observed in mRNA levels of these genes between the two groups. Together, the present study first indicated that transcriptions of *AVPR1a*, *OXTR*, and *PKCB* were regulated by DNA methylation in human umbilical vessels and revealed that hyper-methylation in *AVPR1a*, *OXTR*, and *PKCB* gene promoter, leading to a relatively low pattern of gene expressions, were responsible for the decreased sensitivity of AVP and OXT in pre-eclamptic umbilical vessels. Large amount of research showed that DNA methylation has been considered for contribution to the development for PE [28]. In the present study, we first demonstrated that DNA methylation-mediated gene expression was also critically involved in the pathogenesis of vascular dysfunction in pre-eclamptic umbilical vasculature. The pathological and clinical importance of DNA methylation in pre-eclamptic vascular dysfunction deserves further investigation.

Significance of our findings is also closely linked to “the development of chronic diseases in fetal origins.” According to this theory, prenatal adverse factors have been demonstrated as major causes to induce diseases after birth [29–31]. PE could act as a stress for development fetuses. Thirty percent of newborns born from pre-eclamptic women experience some forms of adverse prenatal outcome, including prematurity and intrauterine growth retardation [1, 2]. PE is a long-term disease risk factor for the mother and possibly the offspring too [32–34]. Evidence from clinical studies has proposed that children born from pre-eclamptic women have a higher risk of suffering neurological, psychological, and behavioral alterations, particularly cardiovascular diseases, including hypertension and stroke, compared to children born from normal pregnancies [32, 33, 35–40]. However, to date, the mechanisms behind these vascular outcomes are poorly understood. In human, the umbilical cord is physiologically and genetically part of the fetus and may reflect problems originated from inadequate changes in the fetus with maternal history of PE. Interestingly, this study found that pre-eclamptic fetal umbilical vein showed a specific epigenetic-mediated vascular dysfunction, suggesting that pre-eclamptic fetal vascular system may undergo similar changes as it is represented in fetal umbilical vessels. It is rational that there may exist the same or similar abnormalities in pre-eclamptic fetal vascular systems as that observed in the fetal umbilical cord vein. Given this, due to epigenetic code that can be inherited, it put forward the hypothesis that the child with maternal history of PE are with a higher risk of diseases and disorders particularly in vascular problems. Recent cohort studies assessing whether maternal PE are associated with vascular problems in the offspring throughout childhood and early adolescence have provided supportive evidence for this concept [39–42]. Although this study did not investigate the offspring, the interesting finding in epigenetic-mediated umbilical vascular dysfunctions provides new important information for further studies on cardiovascular diseases in fetal origins.

In conclusion, this study firstly revealed a special feature of vascular regulations and pathophysiological changes in the umbilical vein under conditions of PE. Significances of the finding includes (1) offering new information for further understanding the pathological features of pre-eclamptic fetal umbilical vessels, and (2) underlining roles of epigenetic-mediated gene expression in pre-eclamptic umbilical vascular dysfunction, and (3) providing new insights into the underlying mechanisms of PE-related higher risks of vascular diseases and disorders in fetal origins. In addition, it is well known that an altered placenta-umbilical cord circulation plays an important role in the development of PE [2, 43]. Whether and how the changed umbilical vascular dysfunction contributes or complicates to PE is another interesting and important direction for researching.

## Materials and methods

### Sample preparation

Healthy normal pregnant (*N* = 42) and pre-eclamptic women (*N* = 40) were recruited from the local hospitals, Suzhou, China. The Ethics Committee of the First Hospital of Soochow University approved all procedures in this work (ref. no. 2015-129), and all participants were given informed consent. Healthy pregnant participants were defined as blood pressure < 120/90 mmHg and no clinically significant complications. Pre-eclamptic pregnant participants were defined as blood pressure > 140/90 mmHg and significant proteinuria after the 20th weeks of pregnancy [1, 44]. Women with essential hypertension or medical complications, such as diabetes and renal and cardiovascular diseases, were excluded from the study. The clinical characteristics of all participants were detailed in Table 6.

**Table 6:** Basic characteristics of pre-eclampsia cases and normotensive controls

Characteristics	NP	PE
Number of subjects	42	40
Maternal age (year)	28.40 ± 4.50	28.20 ± 4.10
Gestational age (week)	38.4 ± 2.1	33.3 ± 4.1**
Birth weight (kg)	3.2 ± 0.8	2.6 ± 0.8*
Systolic BP (mmHg)	107.6 ± 7.9	164.8 ± 19.2**
Diastolic BP (mmHg)	79.5 ± 9.8	105.4 ± 12.1**
Proteinuria (g/24 h)	0.17 ± 0.05	5.01 ± 2.62**
S/D ratio	2.02 ± 0.44	3.82 ± 1.86*

The data was expressed as mean ± SD. Pre-eclampsia vs. normal pregnant. S/D ratio, ratio of systolic and diastolic blood flow in the umbilical artery. *PE* pre-eclampsia, *NP* normal pregnant. **P < 0.01; ***P < 0.001

### Measurement of vascular tension

Human umbilical cords were immediately acquired from normal and pre-eclamptic pregnant women after vaginal delivery and delivered within 1 h. The umbilical cords (10 cm in length) were kept in iced Krebs solution (containing in mmol/L NaCl 119, NaHCO_3_ 25, KH_2_PO_4_ 1.2, KCl 4.7, MgSO_4_ 1.0, glucose 11, and CaCl_2_ 2.5), and bubbled with 95% O_2_ and 5% CO_2_. Human umbilical vein was carefully isolated and cut into rings approximately 4–5 mm in length and suspended in a 5 mL organ bath with 5 mL Krebs solution and continuously with a mixture of 95% O_2_ and 5% CO_2_. Under the tension of 2 g, HUV rings were allowed to balance for 2 h. Then the contraction of potassium chloride (KCl, 120 mol/L) was used to gain maximum vascular reaction. The contraction induced by AVP or OXT was standardized through comparing with the maximal tension caused by KCl. HUV rings were contracted by the addition of incremental doses of vasopressin AVP (10^−10^ to 10^−4^ mol/L), OXT (10^−10^ to 10^−4^ mol/L), or PDBu (Phorbol 12, 13-dibutyrate, PKC activator; 10^−10^ to 10^−5^ mol/L) at 4-min intervals. Between continuous concentrations of AVP, OXT, or PDBu, there was at least 4 min of reaction time, during that period, the reaction of preceding concentration reached equilibrium phase. In the subsequent experiment, SR49059 (a specific inhibitor of AVP, 10 μmol/L), atosiban (a specific inhibitor of OXT, 10 μmol/L), or GF109203X (PKC antagonist, 100 μmol/L) were used for pretreating HUV rings for 30 min before application of AVP or OXT, and vessel responses were recorded [45]. All drugs were freshly prepared and purchased from Sigma-Aldrich (St. Louis, MO).

### Quantitative real-time PCR (qRT-PCR) and Western blot analysis

Total RNA was isolated from HUV using Trizol reagent and was reversed transcribed using first-strand cDNA Synthesis Kit (Invitrogen). qRT-PCR was performed using SYBR Green Supermix Taq Kit (Takara Biotechnology Co., Ltd., Dalian, China) and analyzed on an iQ5 Real-Time PCR Detection System (Bio-Rad Laboratories, Inc., Hercules, CA, USA). The primer sequences are listed in Table 7. ∆∆Ct method was used to comparatively quantify the amount of mRNA levels. The protein abundance of AVPR1a, AVPR2, OXTR, and PKC (α and β) in HUV was measured with Western blot normalized to β-actin. The primary antibodies were the rabbit polyclonal antibody (Santa Cruz Biotechnology) against AVPR1a, AVPR2, OXTR, or PKCβ (all 1:1000). The secondary antibody was the goat anti-rabbit antibody (1:500; Beyotime Biotechnology, Jiangsu, China). Immuno-signals were visualized using UVP imaging system (EC3-Imaging-System, Upland, CA, USA). Imaging signals were calculated and analyzed, and then the ratio of band brightness to β-actin was acquired to measure relative protein expression levels as previously described [46, 47].

**Table 7:** List of oligonucleotide primers used in this study

Primer	Nucleotide Sequence (5′ to 3′)	
	Sense	Anti-sense
qRT-PCR primers		
AVPR1a	TCGTGACGGCTTACATCGTC	GAGTCTTGAAGGAGATGGCCA
AVPR1b	CCTCATCTGCCATGAGATCTG	GCCACATTGGTGGAATCTTCATCA
AVPR2	TGACGCTAGTGATTGTGGTC	GACACGCTGCTGCTGAAAGA
OTXR	TCAGCAGCGTCAAGCTCATC	GTGAACAGCATGTAGATCCAG
PKCα	CTCTGCGGAATGGATCACACT	GGACTCATTCCACTGCGGAT
PKCβ	GACCTCATGTATCACATCCAG	GAGTGCCACAGAATGTCTTG
PKCδ	TCCAAGGACATCCTGGAGAAG	GTCTCTGGGTGACTTCACTT
PKCε	TACAAGGTCCCTACCTTCTG	TCGGCCAGTACTTTGGCGAT
PKCγ	TGCCTGTGCCCGTCATATCCT	AGAGTCCAGAACGCTAAGGT
Bisulfite sequencing primers		
AVPR1a	AGAGTTAGGTTTAGGTGTAGGAGTTAGATG	CAAATTCCTCCTCACAATAAATAAAATC
OTXR	TTTYGTTTYGGAGGGGTTTG	AATACTAAACTAAAATCTCTCACTAAAACCTC
PKCβ-1	GGTAGTAGTTGGGYGAGTGATAgttt	ACCCCRCAACCRAATCAAC
PKCβ-2	GgtttYGgggtYGgtATTTTT	CTCACCAAATAAAATCRATACAATAACTACAAA

### DNA isolation and targeted bisulfite sequencing assay

To prepare genomic DNA, HUV rings were lysed with lysis buffer containing (10 mM Tris-Cl (pH 7.5), 10 mM NaCl, 10 mM EDTA, 0.5% sarcosyl, and 1 mg/mL proteinase K) and incubated overnight at 60 °C. Genomic DNA was extracted from lysates by standard phenol/chloroform technique and subjected to bisulfite conversion using EpiTect bisulfite kit (Qiagen) according to the manufacturer’s protocols. DNA was quantified and then diluted to a working concentration of 20 ng/μL for BiSulfite Amplicon Sequencing (BSAS) [48]. CpG islands located in the proximal promoter of *AVPR1a*, *OXTR*, and *PKCB* were selected according to the following criteria: (1) ≥ 200 bp length, (2) ≥ 50% GC content, (3) ≥ 60% ratio of observed/expected dinucleotides CpG. Based on the genomic coordinates of the candidate CpG sites (Table 3, 4, 8), we carefully designed the BSAS primers in order to detect them in a panel (Table 7). After PCR amplification, products were sequenced by Illumina Hiseq 2000. Methylation level at each tested CpG site was calculated as the percentage of the methylated cytosines over the total tested cytosines. The average methylation levels were calculated using methylation levels of all measured CpG sites within the *AVPR1a*, *OXTR*, or *PKCB* gene.

**Table 8:** Methylated CpG sites at *PKCΒ* gene promoter measured in this study

Gene	Position	Genomic location	Relative to TSS, bp	Position	Genomic location	Relative to TSS, bp
*PKCB*	1	Chr16: 23847338	+ 27	23	Chr16: 23847448	137
	2	Chr16: 23847344	+ 33	24	Chr16: 23847450	139
	3	Chr16: 23847346	+ 35	25	Chr16: 23847456	145
	4	Chr16: 23847348	+ 37	26	Chr16: 23847462	151
	5	Chr16: 23847351	+ 40	27	Chr16: 23847472	157
	6	Chr16: 23847353	+ 42	28	Chr16: 23847479	164
	7	Chr16: 23847357	+ 46	29	Chr16: 23847487	172
	8	Chr16: 23847366	+ 55	30	Chr16: 23847489	174
	9	Chr16: 23847369	+ 58	31	Chr16: 23847491	176
	10	Chr16: 23847380	+ 69	32	Chr16: 23847507	192
	11	Chr16: 23847384	+ 73	33	Chr16: 23847513	198
	12	Chr16: 23847386	+ 75	34	Chr16: 23847519	204
	13	Chr16: 23847392	+ 81	35	Chr16: 23847522	207
	14	Chr16: 23847404	+ 93	36	Chr16: 23847525	210
	15	Chr16: 23847408	+ 97	37	Chr16: 23847529	214
	16	Chr16: 23847411	+ 100	38	Chr16: 23847535	220
	17	Chr16: 23847418	+ 107	39	Chr16: 23847547	232
	18	Chr16: 23847420	+ 109	40	Chr16: 23847551	236
	19	Chr16: 23847428	+ 117	41	Chr16: 23847556	241
	20	Chr16: 23847433	+ 122	42	Chr16: 23847560	245
	21	Chr16: 23847440	+ 129	43	Chr16: 23847568	253
	22	Chr16: 23847442	+ 131	44	Chr16: 23847575	260

### Isolation and culture HUVECs

Umbilical cords (about 15 cm in length) were excised from the placenta immediately after delivery and placed into cold sterile phosphate-buffered saline. Endothelial cells were isolated from umbilical veins as described previously [46]. HUVECs were cultured in DMEM containing 20% fetal bovine serum at 37 °C with 5% CO_2_ and 95% air humidified incubator. Cultures were passaged every 2–3 days and used in experiments 2 passages. In 5-Aza treatment studies, HUVECs were seeded and allowed to grow for 2 days with or without 5-Aza (Sigma-Aldrich, 10^−6^ mol/L) and then mRNA were extracted for experiments.

## Data analysis and statistics

All data were expressed as the mean ± SEM. Significance (*P* < 0.05) was ascertained by *t* test or two-way analysis of variance (ANOVA) followed by the Bonferroni test. Concentration-dependent response curves were performed by computer-assisted nonlinear regression. DNA methylation/mRNA correlation plots were identified by causal inference test (Graph Pad Prism software CA, USA).

## Abbreviations

Atosiban: OXT-specific antagonist
AVP: Arginine vasopressin
GF109203X: PKC antagonist
HUV: Human umbilical vein
KCl: Potassium chloride
NP: Normal pregnancies
OXT: Oxytocin
PDBu: Phorbol 12, 13-dibutyrate (PKC activator)
PE: Pre-eclampsia
PKC: Protein kinase C
SR49059: AVPR1a-specific antagonist

## References

[1] Steegers EA, von Dadelszen P, Duvekot JJ, Pijnenborg R (2010). Pre-eclampsia. Lancet.

[2] Chaiworapongsa T, Chaemsaithong P, Yeo L, Romero R (2014). Pre-eclampsia part 1: Current understanding of its pathophysiology. Nat Rev Nephrol.

[3] Walker DW, McLean JR (1971). Absence of adrenergic nerves in the human placenta. Nature.

[4] Reilly FD, Russell PT (1977). Neurohistochemical evidence supporting an absence of adrenergic and cholinergic innervation in the human placenta and umbilical cord. Anat Rec.

[5] Japundzic-Zigon N (2013). Vasopressin and oxytocin in control of the cardiovascular system. Curr Neuropharmacol.

[6] Yang G, Li T, Xu J, Liu L (2010). Pkc plays an important mediated effect in arginine vasopressin induced restoration of vascular responsiveness and calcium sensitization following hemorrhagic shock in rats. Eur J Pharmacol.

[7] Koshimizu TA, Nakamura K, Egashira N, Hiroyama M, Nonoguchi H, Tanoue A (2012). Vasopressin v1a and v1b receptors: from molecules to physiological systems. Physiol Rev.

[8] Koshimizu TA, Nasa Y, Tanoue A, Oikawa R, Kawahara Y, Kiyono Y, Adachi T, Tanaka T, Kuwaki T, Mori T (2006). V1a vasopressin receptors maintain normal blood pressure by regulating circulating blood volume and baroreflex sensitivity. Proc Natl Acad Sci U S A.

[9] Wellmann S, Benzing J, Cippa G, Admaty D, Creutzfeldt R, Mieth RA, Beinder E, Lapaire O, Morgenthaler NG, Haagen U (2010). High copeptin concentrations in umbilical cord blood after vaginal delivery and birth acidosis. J Clin Endocrinol Metab.

[10] Polin RA, Husain MK, James LS, Frantz AG (1977). High vasopressin concentrations in human umbilical cord blood--lack of correlation with stress. J Perinat Med.

[11] de Geest K, Thiery M, Piron-Possuyt G, Vanden Driessche R (1985). Plasma oxytocin in human pregnancy and parturition. J Perinat Med.

[12] Chard T, Hudson CN, Edwards CR, Boyd NR (1971). Release of oxytocin and vasopressin by the human foetus during labour. Nature.

[13] Arrowsmith S, Wray S (2014). Oxytocin: Its mechanism of action and receptor signalling in the myometrium. J Neuroendocrinol.

[14] Yang G, Xu J, Li T, Ming J, Chen W, Liu L (2010). Role of v1a receptor in avp-induced restoration of vascular hyporeactivity and its relationship to mlcp-mlc20 phosphorylation pathway. J Surg Res.

[15] Eto M, Kitazawa T, Brautigan DL (2004). Phosphoprotein inhibitor cpi-17 specificity depends on allosteric regulation of protein phosphatase-1 by regulatory subunits. Proc Natl Acad Sci U S A.

[16] Ringvold HC, Khalil RA (2017). Protein kinase c as regulator of vascular smooth muscle function and potential target in vascular disorders. Adv Pharmacol.

[17] Chen YL, Shepherd C, Spinelli W, Lai FM (1999). Oxytocin and vasopressin constrict rat isolated uterine resistance arteries by activating vasopressin v1a receptors. Eur J Pharmacol.

[18] Okhovat M, Maguire SM, Phelps SM (2017). Methylation of avpr1a in the cortex of wild prairie voles: effects of cpg position and polymorphism. R Soc Open Sci.

[19] Bodden C, van den Hove D, Lesch KP, Sachser N (2017). Impact of varying social experiences during life history on behaviour, gene expression, and vasopressin receptor gene methylation in mice. Sci Rep.

[20] Okhovat M, Chen IC, Dehghani Z, Zheng DJ, Ikpatt JE, Momoh H, Phelps SM (2018). Genetic variation in the developmental regulation of cortical avpr1a among prairie voles. Genes Brain Behav.

[21] Smearman EL, Almli LM, Conneely KN, Brody GH, Sales JM, Bradley B, Ressler KJ, Smith AK (2016). Oxytocin receptor genetic and epigenetic variations: association with child abuse and adult psychiatric symptoms. Child Dev.

[22] Mamrut S, Harony H, Sood R, Shahar-Gold H, Gainer H, Shi YJ, Barki-Harrington L, Wagner S (2013). DNA methylation of specific cpg sites in the promoter region regulates the transcription of the mouse oxytocin receptor. PLoS One.

[23] Kusui C, Kimura T, Ogita K, Nakamura H, Matsumura Y, Koyama M, Azuma C, Murata Y (2001). DNA methylation of the human oxytocin receptor gene promoter regulates tissue-specific gene suppression. Biochem Biophys Res Commun.

[24] Hagiwara K, Ito H, Murate T, Miyata Y, Ohashi H, Nagai H (2012). Prox1 overexpression inhibits protein kinase c beta ii transcription through promoter DNA methylation. Genes Chromosomes Cancer.

[25] Li C, Gao W, Gao Y, Yu C, Lv J, Lv R, Duan J, Sun Y, Guo X (2018). Age prediction of children and adolescents aged 6-17 years: an epigenome-wide analysis of DNA methylation. Aging.

[26] Liu S, Chen X, Chen R, Wang J, Zhu G, Jiang J, Wang H, Duan S, Huang J (2017). Diagnostic role of wnt pathway gene promoter methylation in non small cell lung cancer. Oncotarget.

[27] Heo HJ, Tozour JN, Delahaye F, Zhao Y, Cui L, Barzilai N, Einstein FH (2016). Advanced aging phenotype is revealed by epigenetic modifications in rat liver after in utero malnutrition. Aging Cell.

[28] Yeung KR, Chiu CL, Pidsley R, Makris A, Hennessy A, Lind JM (2016). DNA methylation profiles in preeclampsia and healthy control placentas. Am J Physiol Heart Circ Physiol.

[29] Gao Q, Tang J, Chen J, Jiang L, Zhu X, Xu Z (2014). Epigenetic code and potential epigenetic-based therapies against chronic diseases in developmental origins. Drug Discov Today.

[30] Murphy MO, Cohn DM, Loria AS (2017). Developmental origins of cardiovascular disease: impact of early life stress in humans and rodents. Neurosci Biobehav Rev.

[31] Buffington SA, Di Prisco GV, Auchtung TA, Ajami NJ, Petrosino JF, Costa-Mattioli M (2016). Microbial reconstitution reverses maternal diet-induced social and synaptic deficits in offspring. Cell.

[32] Davis EF, Lazdam M, Lewandowski AJ, Worton SA, Kelly B, Kenworthy Y, Adwani S, Wilkinson AR, McCormick K, Sargent I (2012). Cardiovascular risk factors in children and young adults born to pre-eclamptic pregnancies: a systematic review. Pediatrics.

[33] Alsnes IV, Vatten LJ, Fraser A, Bjorngaard JH, Rich-Edwards J, Romundstad PR, Asvold BO (2017). Hypertension in pregnancy and offspring cardiovascular risk in young adulthood: prospective and sibling studies in the hunt study (nord-trondelag health study) in norway. Hypertension.

[34] Beukers F, Aarnoudse-Moens CSH, van Weissenbruch MM, Ganzevoort W, van Goudoever JB, van Wassenaer-Leemhuis AG. Maternal psychological distress after severe pregnancy hypertension was associated with increased child behavioural problems at the age of 12. Acta Paediatrica. 2018. 10.1111/apa.14676 30506609

[35] Figueiro-Filho EA, Mak LE, Reynolds JN, Stroman PW, Smith GN, Forkert ND, Paolozza A, Ratsep MT, Croy BA (2017). Neurological function in children born to pre-eclamptic and hypertensive mothers - a systematic review. Pregnancy Hypertens.

[36] Lara E, Acurio J, Leon J, Penny J, Torres-Vergara P, Escudero C (2018). Are the cognitive alterations present in children born from pre-eclamptic pregnancies the result of impaired angiogenesis? Focus on the potential role of the vegf family. Frontiers Physiology.

[37] Fields JA, Garovic VD, Mielke MM, Kantarci K, Jayachandran M, White WM, Butts AM, Graff-Radford J, Lahr BD, Bailey KR, Miller VM (2017). Preeclampsia and cognitive impairment later in life. Am J Obstet Gynecol.

[38] Davis EF, Lewandowski AJ, Aye C, Williamson W, Boardman H, Huang RC, Mori TA, Newnham J, Beilin LJ, Leeson P (2015). Clinical cardiovascular risk during young adulthood in offspring of hypertensive pregnancies: insights from a 20-year prospective follow-up birth cohort. BMJ Open.

[39] Bellamy L, Casas JP, Hingorani AD, Williams DJ (2007). Pre-eclampsia and risk of cardiovascular disease and cancer in later life: systematic review and meta-analysis. Bmj.

[40] Kajantie E, Eriksson JG, Osmond C, Thornburg K, Barker DJ (2009). Pre-eclampsia is associated with increased risk of stroke in the adult offspring: The helsinki birth cohort study. Stroke.

[41] Hakim J, Senterman MK, Hakim AM (2013). Preeclampsia is a biomarker for vascular disease in both mother and child: the need for a medical alert system. Int J Pediatr.

[42] Hoodbhoy Z, Hasan BS, Mohammed N, Chowdhury D (2018). Impact of pre-eclampsia on the cardiovascular health of the offspring: a cohort study protocol. BMJ Open.

[43] Gao Q, Tang J, Li N, Liu B, Zhang M, Sun M, Xu Z (2018). What is precise pathophysiology in development of hypertension in pregnancy? Precision medicine requires precise physiology and pathophysiology. Drug Discov Today.

[44] Young BC, Levine RJ, Karumanchi SA (2010). Pathogenesis of preeclampsia. Annu Rev Pathol.

[45] Zhu X, Gao Q, Tu Q, Zhong Y, Zhu D, Mao C, Xu Z (2016). Prenatal hypoxia enhanced angiotensin ii-mediated vasoconstriction via increased oxidative signaling in fetal rats. Reprod Toxicol.

[46] Gao Q, Zhu X, Chen J, Mao C, Zhang L, Xu Z (2016). Upregulation of p53 promoted g1 arrest and apoptosis in human umbilical cord vein endothelial cells from preeclampsia. J Hypertens.

[47] Liu X, Gao Q, Li P, Zhao Q, Zhang J, Li J, Koseki H, Wong J (2013). Uhrf1 targets dnmt1 for DNA methylation through cooperative binding of hemi-methylated DNA and methylated h3k9. Nat Commun.

[48] Masser DR, Berg AS, Freeman WM (2013). Focused, high accuracy 5-methylcytosine quantitation with base resolution by benchtop next-generation sequencing. Epigenetics Chromatin.

